# Dual regulation of water retention and cell growth by a *stress-associated protein* (*SAP*) gene in *Prunus*

**DOI:** 10.1038/s41598-017-00471-7

**Published:** 2017-03-23

**Authors:** Alba Lloret, Ana Conejero, Carmen Leida, César Petri, Francisco Gil-Muñoz, Lorenzo Burgos, María Luisa Badenes, Gabino Ríos

**Affiliations:** 1 Instituto Valenciano de Investigaciones Agrarias (IVIA), 46113 Moncada, Valencia Spain; 2 Department of Plant Production, Instituto de Biotecnología Vegetal-Universidad Politécnita de Cartagena (IBV-UPCT), 30202 Cartagena, Murcia Spain; 3 Group of Fruit Tree Biotechnology, Department of Plant Breeding, CEBAS-CSIC, 30100 Murcia, Spain

## Abstract

We have identified a gene (*PpSAP1*) of *Prunus persica* coding for a stress-associated protein (SAP) containing Zn-finger domains A20 and AN1. SAPs have been described as regulators of the abiotic stress response in plant species, emerging as potential candidates for improvement of stress tolerance in plants. *PpSAP1* was highly expressed in leaves and dormant buds, being down-regulated before bud dormancy release. *PpSAP1* expression was moderately induced by water stresses and heat in buds. In addition, it was found that PpSAP1 strongly interacts with polyubiquitin proteins in the yeast two-hybrid system. The overexpression of *PpSAP1* in transgenic plum plants led to alterations in leaf shape and an increase of water retention under drought stress. Moreover, we established that leaf morphological alterations were concomitant with a reduced cell size and down-regulation of genes involved in cell growth, such as *GROWTH-REGULATING FACTOR* (*GRF*)1-like, *TONOPLAST INTRINSIC PROTEIN* (*TIP*)-like, and *TARGET OF RAPAMYCIN* (*TOR*)-like. Especially, the inverse expression pattern of *PpSAP1* and *TOR*-like in transgenic plum and peach buds suggests a role of PpSAP1 in cell expansion through the regulation of TOR pathway.

## Introduction

Perennial plants in temperate climates have to cope with seasonal fluctuations in temperature. Particularly, during the winter period they deal with the deleterious effects of cold and water stresses by stopping growth and protecting their dormant meristems into specialized buds. In many cases, proper bud dormancy release and growth resumption requires a quantitative perception of chilling by a yet unknown mechanism, which in certain aspects resembles the vernalization process described in *Arabidopsis* and cereals^1–3^.

We have previously characterized transcriptomic changes associated with dormancy release in reproductive buds of peach (*Prunus persica* [L.] Batsch)^4, 5^. A gene coding for a protein with AN1 and A20 Zn-finger domains has been consistently found to be up-regulated in dormant buds in these studies. Interestingly, gene expression down-regulation occurs concomitantly with dormancy release in genotypes with different chilling requirements, and thus gene expression regulation seems to associate with the developmental stage of buds under apparently variable environmental circumstances^4^. This Zn-finger protein belongs to a family of plant regulators known as stress-associated proteins (SAP), with known homologs in animals^6, 7^.

*SAP* genes have been related to the abiotic stress response in plants. Most commonly, *SAP* genes have been found to be up-regulated under a combination of stressing conditions, including high temperature^8^, chilling^9^, osmotic stress and salinity^10^, water deficit^11^, and heavy metals^12^, among others. In addition, when overexpressed in transgenic plants, *SAP* genes confer tolerance to abiotic stresses^13–16^.

In spite of the numerous studies devoted to *SAP* genes in plants, little is known about their molecular function. AtSAP5 from *Arabidopsis* binds different linkage-specific polyubiquitin chains but not monoubiquitin^17^ and shows E3 ubiquitin ligase activity^10^. The tumor suppressor c-myc binding protein (MBP-1) has been identified as an ubiquitination substrate of AtSAP5, which is thus targeted for ubiquitin-dependent proteasome degradation^18^. Regarding stress tolerance, the related OsSAP1 and OsSAP11 from rice interact with the receptor-like kinase OsRLCK253, which in turn confers tolerance to salt and water deficit stress in transgenic *Arabidopsis* plants^19^. Recently, OsSAP1 has been found to interact with an aminotransferase (OsAMTR1) and a pathogenesis-related protein (OsSCP) involved in salt and water stress tolerance pathways^20^. Moreover, a conformational change in response to redox conditions has been observed in AtSAP12 from *Arabidopsis*, which could thus behave as a sensor and transmitter of redox imbalances triggered by different stresses^21^.

We have characterized *PpSAP1* gene expression in different tissues and environmental conditions, and have performed a yeast two-hybrid screening for the identification of putative protein interactors. In order to get deeper insight into SAP function we overexpressed *PpSAP1* in transgenic plum (*Prunus domestica* cv. Claudia Verde), leading to intriguing evidences about a dual role of *PpSAP1* in stress and developmental issues.

## Results

### Identification of a Zn-finger gene developmentally regulated in flower buds of peach

**Figure 1:**
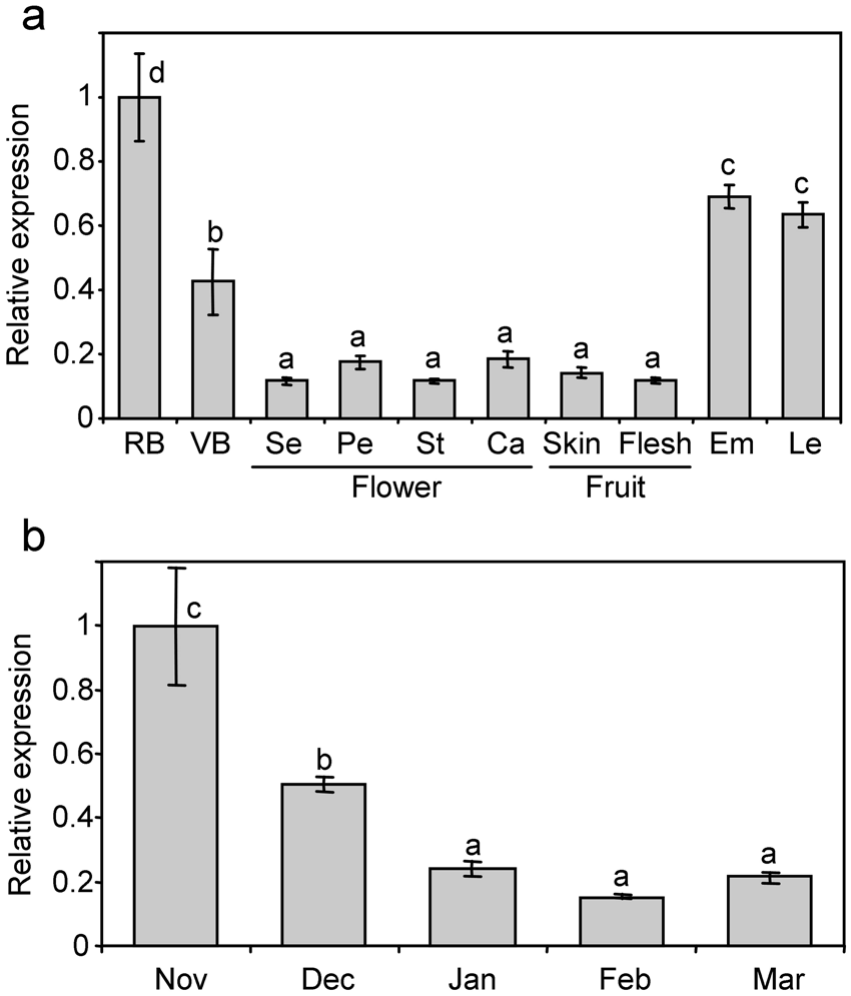
Relative expression of *PsSAP1* in peach by qRT-PCR. In (**a**), different plant tissues were tested, including reproductive bud (RB), vegetative bud (VB), sepal (Se), petal (Pe), stamen (St), carpel (Ca), fruit skin, fruit flesh, embryo (Em) and leaf (Le). In (**b**), reproductive buds were collected at different developmental stages, from November to March. In March samples bud dormancy was already released. An expression value of one is assigned to the first sample. Data are means from two biological samples with three technical replicates each, with error bars representing standard deviation. Different letters (**a**–**d**) indicate significant difference between samples with a confidence level of 95%.

In previous transcriptomic studies in our group we have identified a Zn-finger protein gene expressed in dormant flower buds of peach, which is down-regulated concomitantly with developmental processes leading to bud dormancy release^4, 5^. Formerly known as unigene PpB19^4^, the International Peach Genome Initiative^22^ assigned to this gene model the systematic names ppa012373m (v1.0) and Prupe.2G010400 (v2.1). When analyzing the tissue-dependent expression of ppa012373m we found higher values in reproductive and vegetative buds, embryos and leaves; whereas the different flower and fruit tissues showed lower expression levels (Fig. 1a). As stated in previous reports, its expression decreased along flower bud development in ‘Big Top’ cultivar (Fig. 1b). Interestingly, ppa012373m expression reached its lowest level in January and February samples, previous to bud dormancy release date which was experimentally estimated between February and March sampling dates. Thus, ppa012373m expression in ‘Big Top’ confirmed previous data about its developmental down-regulation in buds, even if it was not tightly associated with bud dormancy release events.

**Figure 2:**
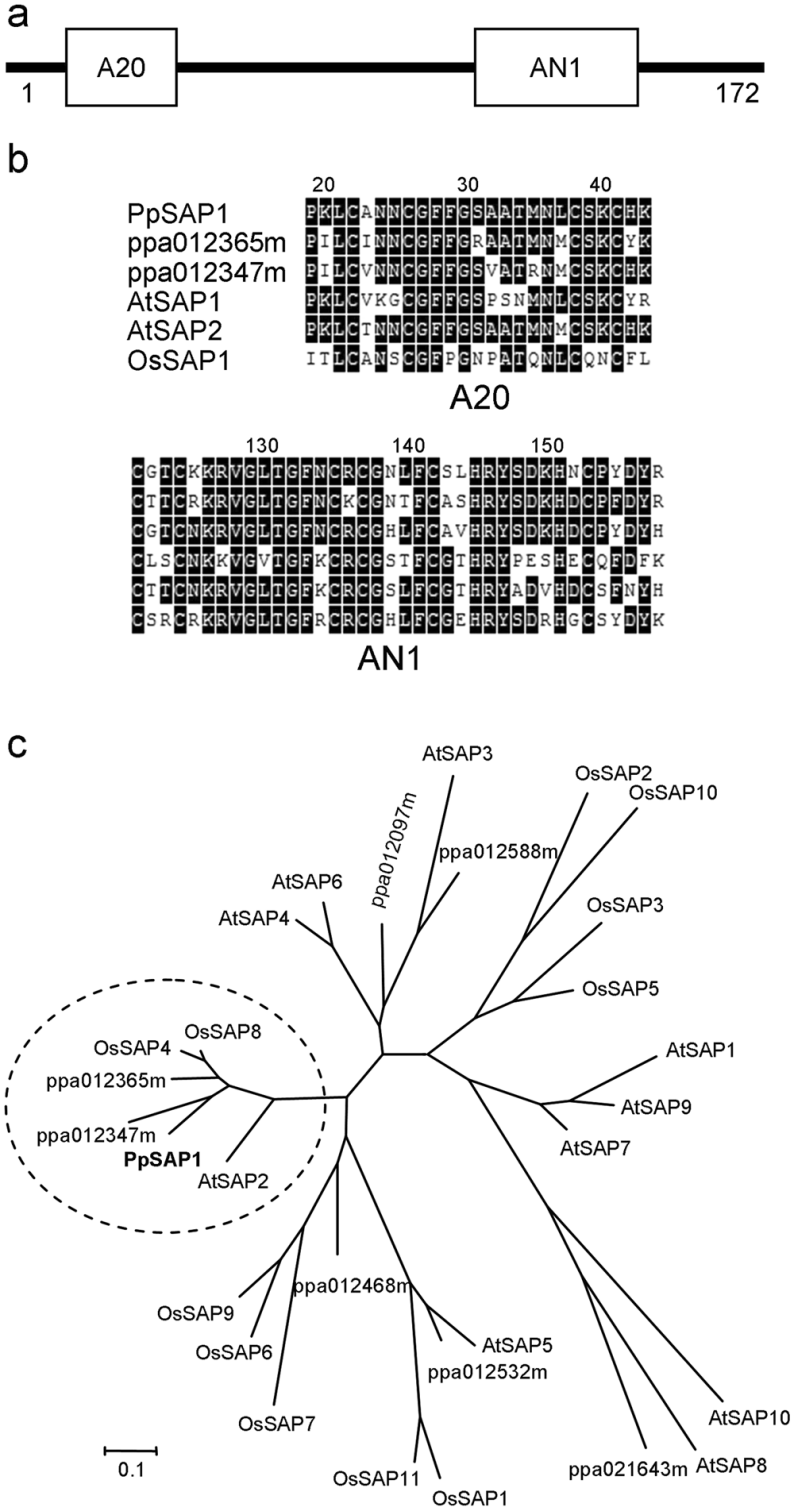
PpSAP1 is a stress-associated protein (SAP). Schematic representation of A20 and AN1 domains in PpSAP1 protein (**a**). Alignment of A20 and AN1 domains from PpSAP1 and other SAP-like proteins of peach, *Arabidopsis* and rice (**b**). In (**c**), phylogenetic tree of SAP proteins from *Arabidopsis*, rice and peach. The tree was constructed using the Maximum Likelihood method and bootstrapped with 1000 replicates. The scale bar indicates the branch length that corresponds to the number of substitutions per amino acid position.

The ppa012373m deduced protein contained two consecutive Zn-finger domains named A20 and AN1 (Fig. 2a,b), which are found together in many stress-associated proteins (SAP) from different plant species. SAP proteins from peach, *Arabidopsis* and rice showing this particular arrangement of A20 and AN1 domains were compared by a phylogenetic analysis. The protein encoded by ppa012373m clustered jointly with two additional peach proteins, *Arabidopsis* AtSAP2, and rice OsSAP4 and OsSAP8, into a group of highly related sequences (Fig. 2c). In virtue of such phylogenetic closeness, from now on we will use the name *PpSAP1* to designate ppa012373m gene.

### *PpSAP1* expression is modulated by abiotic stresses

**Figure 3:**
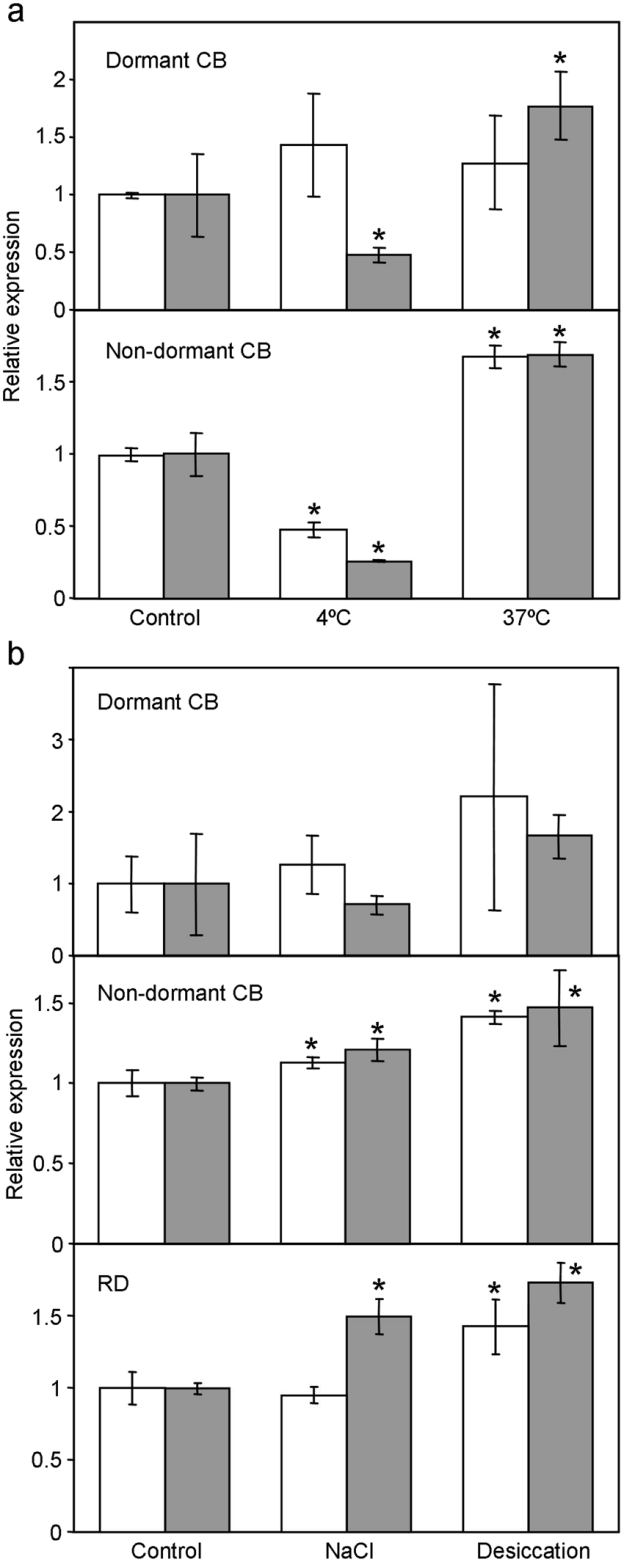
Effect of abiotic stresses on *PpSAP1* expression in peach buds. Treatments at 4 °C and 37 °C (**a**), and NaCl 200 mM and desiccation (**b**) were performed during one (white bars) and three days (grey bars). Dormant and non-dormant reproductive buds from cultivar ‘Crimson Baby’ (CB) and non-dormant buds from ‘Rose Diamond’ (RD) were employed. An expression value of one is assigned to the control. Data are means from three biological samples with two technical replicates each, with error bars representing standard deviation. An asterisk indicates significant difference with the control at a confidence level of 95%.

Often, the expression of *SAP* genes from different species has been found to be induced by environmental cues, mostly abiotic stresses. In order to check the response of *PpSAP1* to abiotic stresses, flowers buds of peach were exposed to temperature and water stresses during one and three days treatments. *PpSAP1* expression was down-regulated by chilling (4 °C) and up-regulated by heating (37 °C) in both dormant and dormancy-released buds, although dormant buds required a longer period of three days to reach a significant difference (Fig. 3a). Water stress induced by desiccation and salinity treatments (NaCl 200 mM) also up-regulated *PpSAP1* expression in non-dormant buds in two different cultivars (Fig. 3b).

On the contrary, different experiments performed in detached leaves and leaf discs did not provide conclusive evidences about an effect of abiotic stresses on *PpSAP1* expression in plant tissues other than buds; made under conditions of desiccation, low temperature and NaCl and abscisic acid incubation that indeed induced strongly the expression of a *LATE EMBRYOGENESIS ABUNDANT* (*LEA*)-like gene (Supplementary Fig. S1).

### PpSAP1 binds to ubiquitin-like proteins

**Figure 4:**
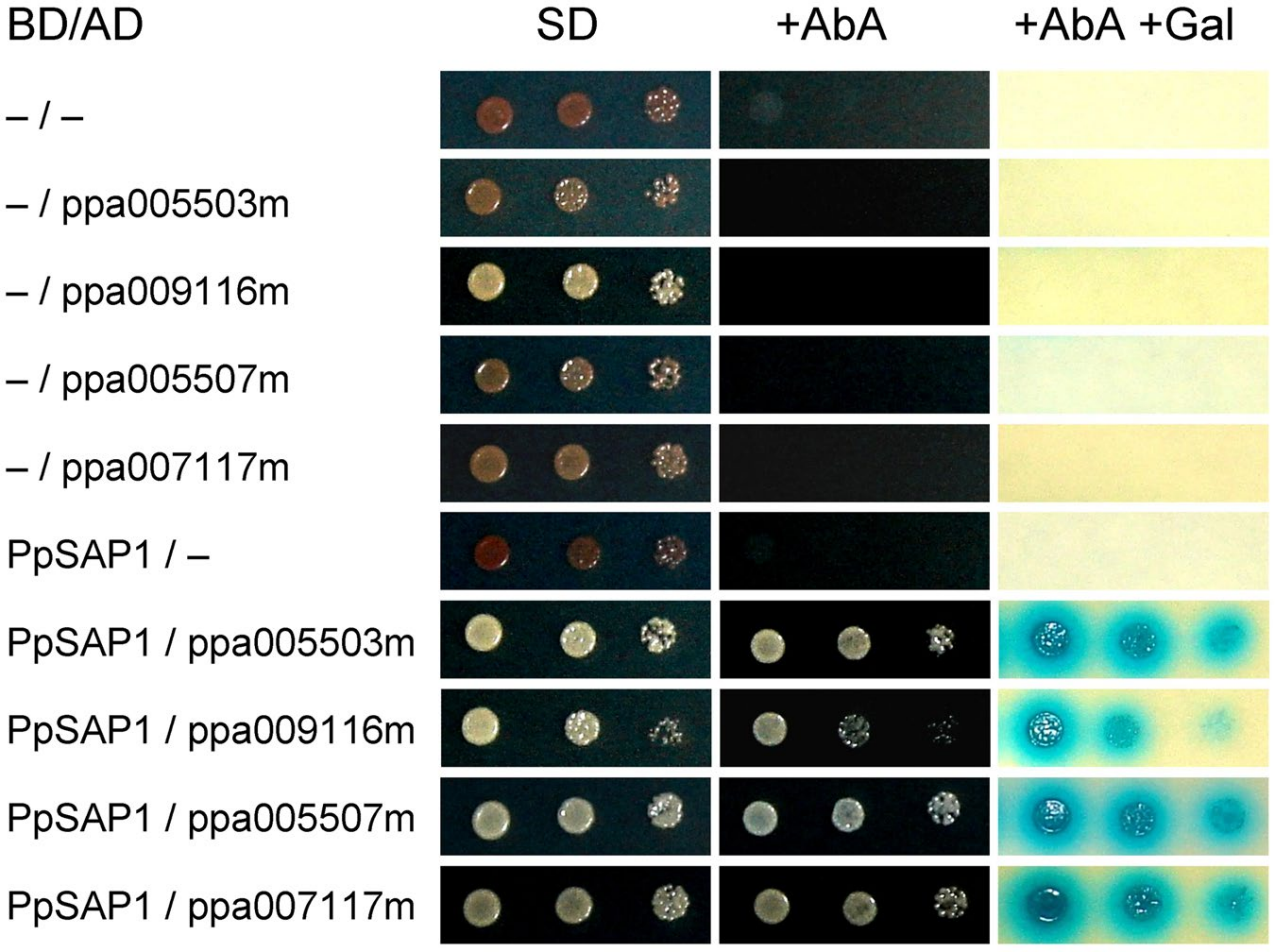
Two-hybrid system analysis of protein interaction. Different combinations of DNA-binding domain (BD) and activation domain (AD) fused with PsSAP1, ppa005503m, ppa009116m, ppa005507m and ppa007117m, and control plasmids (−) are shown. Yeast strains were grown on a minimal medium (SD), a growth selective medium containing Aureobasidin A (+AbA) and a chromogenic medium containing Aureobasidin A and X-α-Gal (+AbA +Gal).

In order to get deeper insight into *PpSAP1* function we performed a yeast-two hybrid screening for the identification of PpSAP1 protein partners and/or targets. *PpSAP1* was cloned into pGBKT7 plasmid as a fusion with the DNA binding domain of GAL4 (BD). This construct was combined by yeast mating with a cDNA library from flower buds of peach into pGADT7 vector expressing the activation domain of GAL4 (AD). We obtained 304 positive colonies that after discarding repeated inserts and false positives were reduced to four independent genes (ppa005503m, ppa009116m, ppa005507m and ppa007117m) coding for polyubiquitin peptides (Fig. 4). This result supported the functional closeness of PpSAP1 to other SAP proteins from plants and animals. The sequence fragments of the positive clones are shown in Supplementary Fig. S2.

### The constitutive expression of *PpSAP1* affects water loss under hydric stress

**Figure 5:**
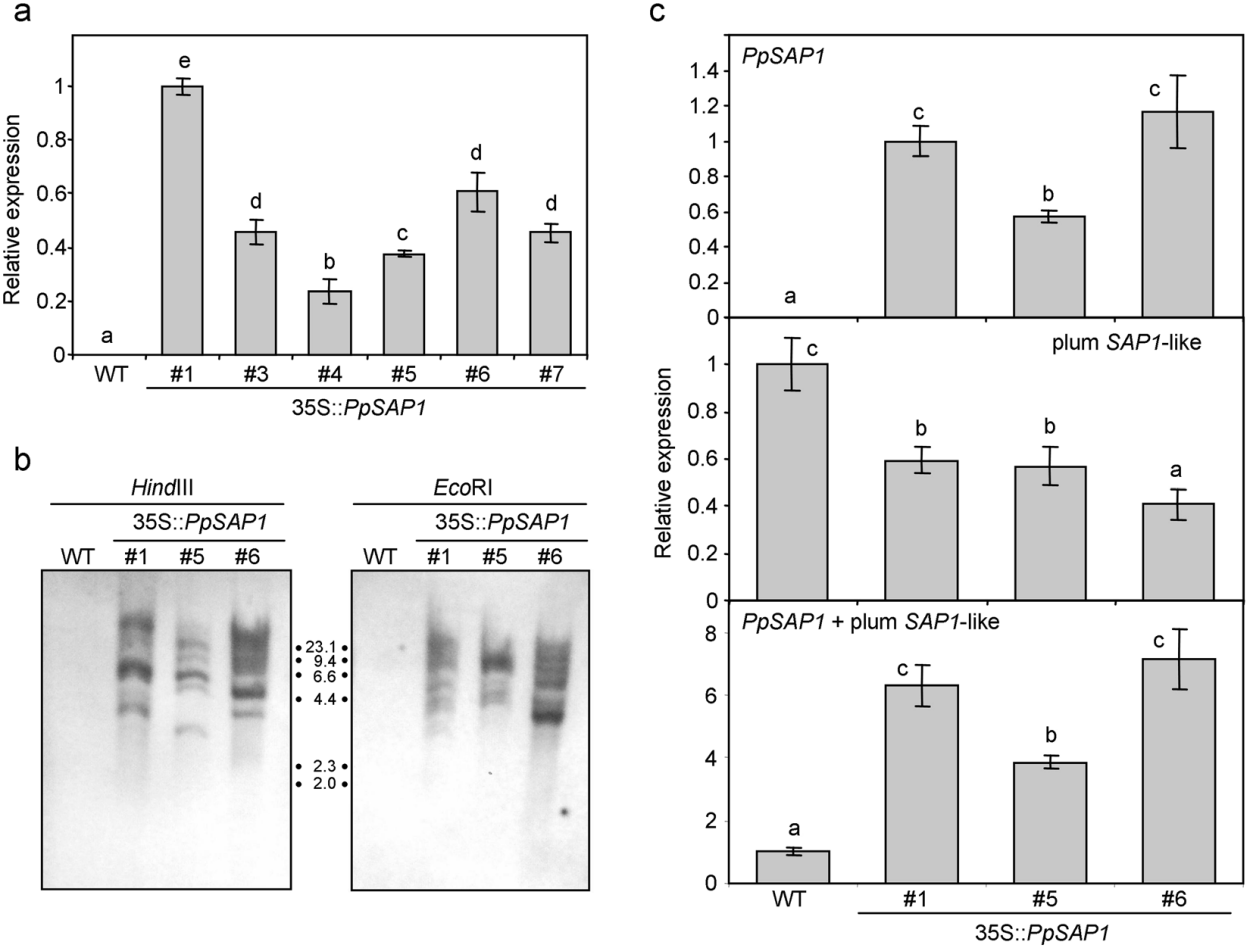
Overexpression of *PpSAP1* in transgenic plum. In (**a**), heterologous expression of *PpSAP1* in six independent transgenic lines of plum (35S::*PpSAP1* #1, #3, #4, #5, #6, #7) and the wild type (WT). Southern analysis with restriction enzymes *Hind*III and *Eco*RI of WT and transgenic lines #1, #5, #6 (**b**), showing the position of molecular weight markers (kb). In (**c**), the relative expression of *PpSAP1*, plum *SAP1*-like and both genes (*PpSAP1* + plum *SAP1*-like) is shown for three transgenic lines, by using specific primers. An expression value of one is assigned to the WT or the transgenic line #1. Data are means from three biological samples with two technical replicates each, with error bars representing standard deviation. Different letters (**a**–**e**) indicate significant difference between samples with a confidence level of 95%.

*PpSAP1* gene was cloned into the binary vector pROK2 for its constitutive expression in plum driven by the 35S promoter. The plum model offered some advantages over other species for gene transformation, including its taxonomical proximity to peach (*Prunus persica*), their common woody perennial habit, similar developmental and physiological issues, and the availability of reliable methods for gene transformation and regeneration^23^. The expression of transgenic *PpSAP1* was assayed in shoots regenerated *in vitro* from six independent plum lines. The six lines expressed *PpSAP1* at varying levels (Fig. 5a). We selected lines #1, #5 and #6 for subsequent analyses. Southern analyses of these lines with two different restriction enzymes revealed the presence of multiple inserts with different integration patterns, confirming their independent origin (Fig. 5b). Once acclimatized, the expression of *PpSAP1*, a plum *SAP1*-like gene and both genes combined was evaluated in these three transgenic lines and the wild type ‘Claudia Verde’ (WT) using specific and common primer pairs (Supplementary Table S1). Leaves from the lines #1 and #6 accumulated more *PpSAP1* transcript than #5, even though its expression level was very high in the three lines and contributed to most of the combined expression of *PpSAP1* plus plum *SAP1*-like (Fig. 5c). On the other hand, the expression of the plum *SAP1*-like ortholog was reduced in the transgenic plants, suggesting the intervention of gene silencing mechanisms.

**Figure 6:**
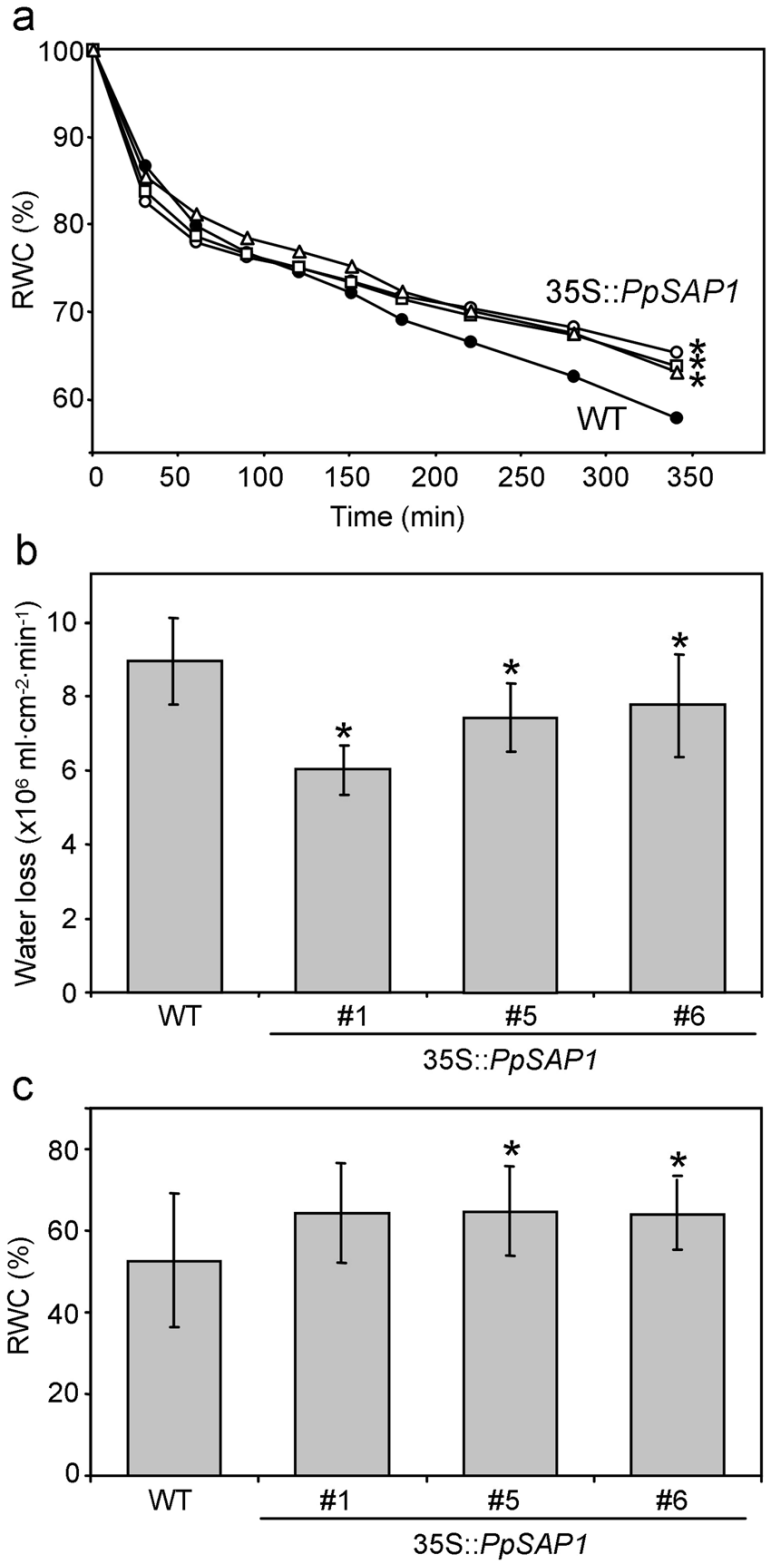
Analysis of water retention in plum overexpressing *PpSAP1* under drought stress. The relative water content (RWC) of leaves detached from wild type (WT, black circles), and 35S::*PpSAP1* lines #1 (white circles), #5 (white squares) and #6 (white triangles) was calculated at different times along the desiccation process (**a**). Data are means from seven plants per genotype, and two leaves per plant. In (**b**), the specific water loss was calculated as the volume of water evaporated per cm^2^ of leaf area and minute, during the time in which evaporation was constant with time in the experiment shown in (**a**). In (**c**), the RWC of whole plants under drought stress for seven days is shown. Data are means from twelve different plants per genotype. Error bars represent standard deviation. An asterisk indicates significant difference with the control at a confidence level of 95%.

*SAP* genes are well known factors of tolerance to abiotic stresses when expressed in heterologous systems. The ability of *PpSAP1* to confer tolerance to abiotic stresses was assessed in overexpressing lines #1, #5 and #6. In a water loss experiment performed in detached leaves, *PpSAP1* overexpressing lines retained higher content of water than WT during the first hours of desiccation (Fig. 6a). In order to determine if such observation was due to differences in the leaf area, we calculated the specific water loss in the range of time in which water loss was lineal. Specific water loss per unit of time and leaf area was also significantly lower in transgenic lines (Fig. 6b), which confirms that differences in relative water content (RWC) were not caused by the distinct size of control and transgenic leaves.

A drought experiment was also performed in whole plants. *PpSAP1* overexpressing lines also retained a higher amount of water after seven days of stress, even though differences were significant in lines #5 and #6 exclusively (Fig. 6c, Supplementary Fig. S3). Additional salinity (NaCl) and heat stress experiments performed in acclimatized and *in vitro* plants did not support significant differences between WT and transgenic lines, thus *PpSAP1* contribution to stress tolerance was limited to drought stress.

### Anatomical and cellular effects of *PpSAP1* expression in transgenic plum

**Figure 7:**
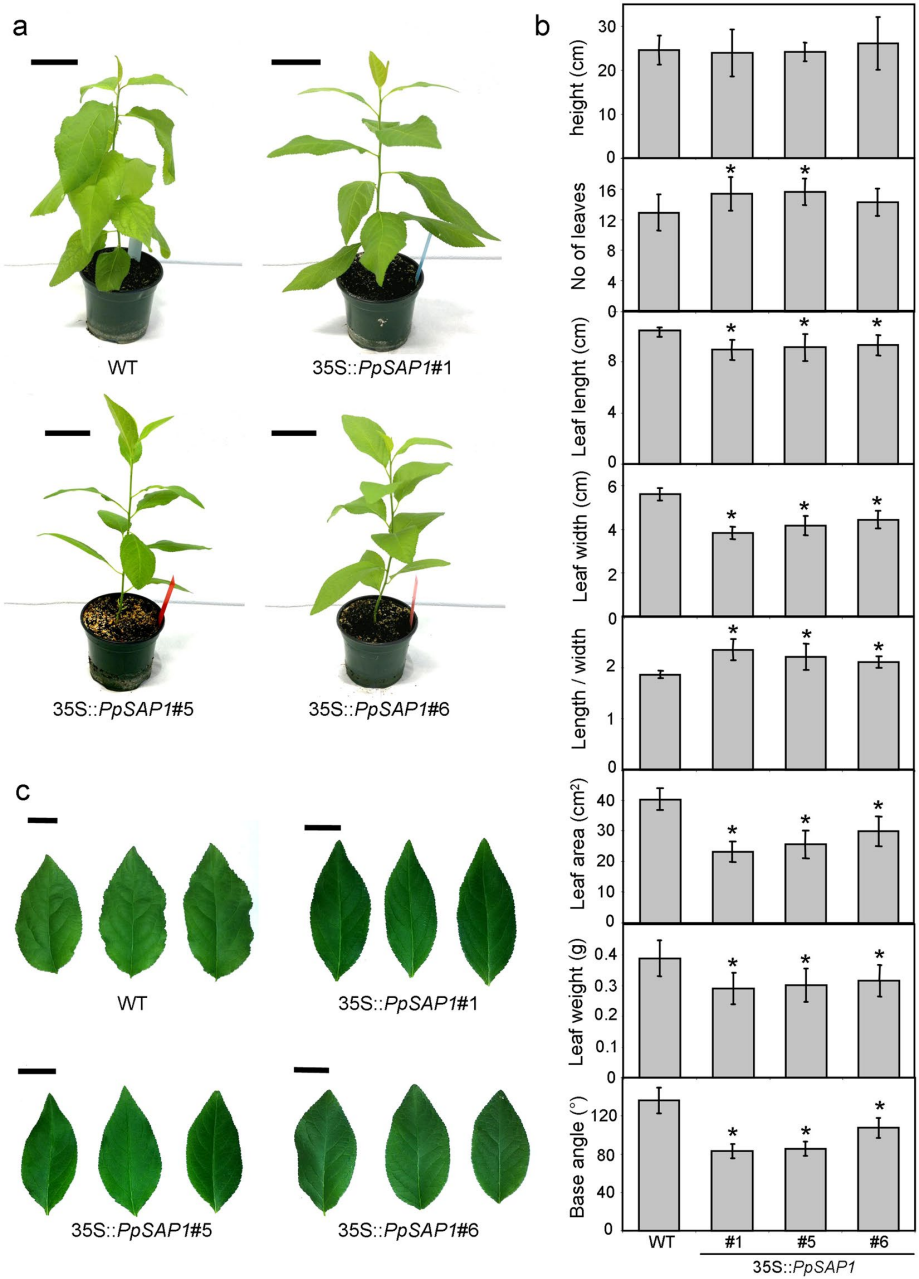
Plant anatomy and leaf morphology in plum overexpressing *PpSAP1*. Two month old plants of wild type (WT) and transgenic lines 35S::*PpSAP1* #1, #5 and #6 are shown (**a**); scale bar, 5 cm. In (**b**), different whole plant and leaf shape parameters of two month old plants are shown. Data are means from twelve different plants, with error bars representing standard deviation. An asterisk indicates significant difference with the control at a confidence level of 95%. Photographic images of detached leaves are shown (**c**); scale bar, 2.5 cm.

The overexpression of *PpSAP1* caused evident effects on leaf morphology and plant growth in the transgenic plums under study: leaves of lines #1, #5 and #6 were smaller and with smoother (less undulate) margins, leading to plants with less dense canopy (Fig. 7a). *PpSAP1* overexpressing plums had a plant height similar to the control, but produced a higher average number of leaves (Fig. 7b). In addition transgenic leaves were shorter, narrower, smaller and lighter, and were different in shape. They had a higher length/width ratio and an acute leaf base angle (Fig. 7b), which caused a change in leaf shape from ovate (control) to elliptical (Fig. 7c).

**Figure 8:**
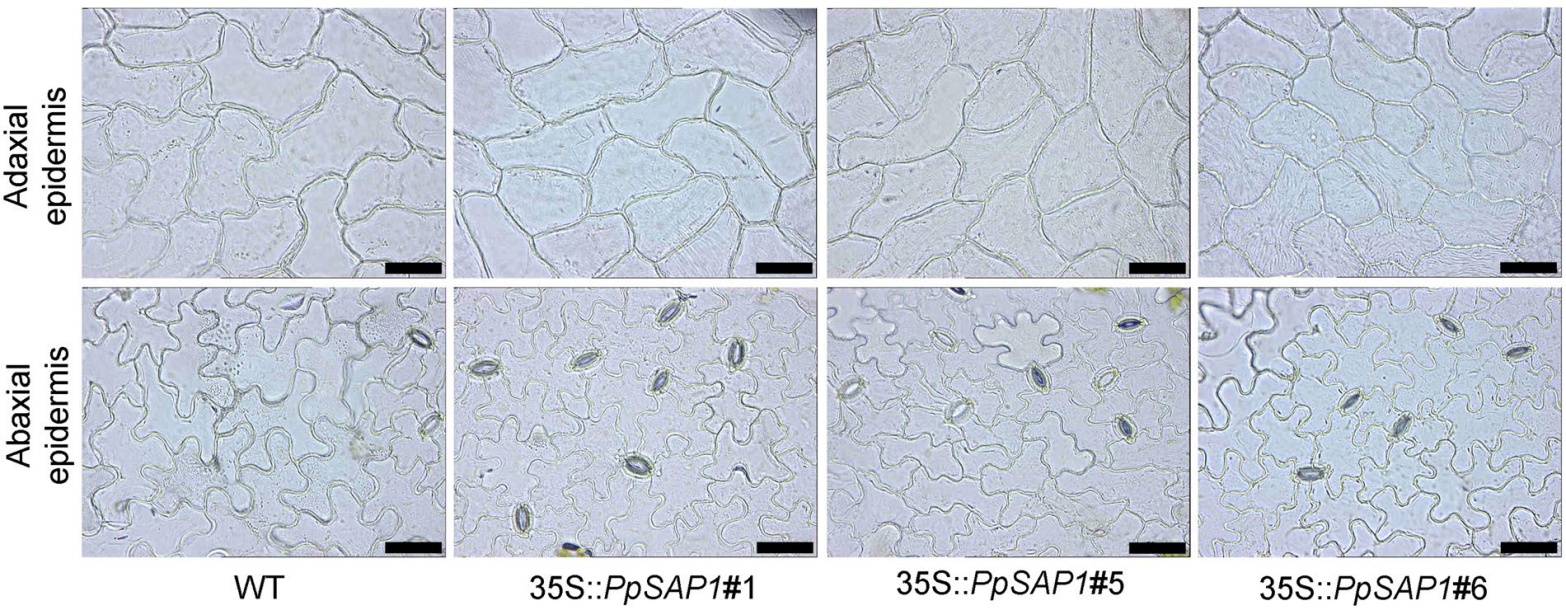
Microscopic photographs of epidermic cells in *PpSAP1* overexpressing lines. The adaxial and abaxial epidermis of wild type (WT) and 35S::*PpSAP1* lines #1, #5 and #6 is shown; scale bar, 50 μm.

Epidermic cells were observed microscopically and their dimensions measured (Fig. 8). Differences in leaf size were associated with the presence of smaller cells in the adaxial and abaxial epidermis of *PpSAP1* overexpressing lines, whereas the calculated number of cells per leaf was not thoroughly reduced in those lines (Table 1). The total number of stomata was similar in control and *PpSAP1* plants, thus leading to an increased density of stomata in the smaller leaves of lines #1, #5 and #6 (Table 1).

**Table 1:** Cell size and number in leaves of transgenic plum overexpressing *PpSAP1*.

	Adaxial epidermis		Abaxial epidermis		Stomata	
	Cell area (μm^2^)	Cell number per leaf (×10^4^)	Cell area (μm^2^)	Cell number per leaf (×10^4^)	Density (mm^−2^)	Number per leaf (×10^4^)
WT	4730 ± 1390	65.4 ± 16.3	3020 ± 1020	102.0 ± 21.5	65.0 ± 13.1	19.3 ± 4.0
35S::*PpSAP*#1	3890 ± 1050**	52.9 ± 8.0*	2550 ± 860**	81.4 ± 17.3*	83.4 ± 14.3**	17.2 ± 4.4
35S::*PpSAP*#5	4040 ± 1090**	53.6 ± 8.1*	2560 ± 1050**	84.6 ± 12.1	79.1 ± 16.6*	16.8 ± 3.6
35S::*PpSAP*#6	3190 ± 1010**	76.7 ± 15.0*	2190 ± 860**	111.0 ± 25.7	87.0 ± 12.8**	20.7 ± 4.8

The significant difference with respect to WT is labelled, with a confidence level of 95% (*) and 99% (**).

### Genes related to cell growth are down-regulated in plum plants overexpressing *PpSAP1*

A series of plum candidate genes to mediate the phenotypical features observed in *PpSAP1* overexpressing plants were selected for quantitative real-time PCR (qRT-PCR) analysis. Thus, several rice and *Arabidopsis* genes showing down- or up-regulated expression in water-deficit stress tolerant plants overexpressing different *SAP* genes^10, 16, 19^ were compared with the peach genome by similarity searches (Supplementary Table S2). We found putative orthologs in peach of nine of these genes by reciprocal BLASTP analysis. In addition, four genes related to drought and stress response identified as differentially regulated in peach buds^5^ were selected for expression analysis (Supplementary Table S2). We designed specific primers for gene expression analysis based on peach sequences. Subsequently, PCR products amplified with such primers using plum cDNA as template were sequenced to confirm that qRT-PCR signals were in fact proceeding from plum putative orthologs of those genes. Among others, we analyzed the expression of late embryogenesis abundant (LEA)-like (ppa008651m), AWPM-19-like (ppa012188m), dehydrin (Prupe.7G161100), ABI5 binding protein (ppa006974m), histone H1-3 (ppa011941m), galactinol synthase 2 (ppa008294m), NaCl-inducible calcium-binding protein (ppa012594m), and responsive to desiccation (RD)29B (ppa001989m). However, none of them showed an altered pattern of expression in *PpSAP1* overexpressing lines (Supplementary Fig. S4). Thus, transcriptional targets of *PpSAP1* could be different from targets described for *SAP* genes from *Arabidopsis* and rice, or alternatively the observed effect on water retention could rely on the regulation of protein stability or activity instead of transcriptional regulation.

In parallel, a similar approach to identify putative orthologs in plum by successive reciprocal BLASTP analysis in peach and sequencing of plum amplicons was applied to several *Arabidopsis* genes involved in leaf morphology and cell growth (Supplementary Table S2).

**Figure 9:**
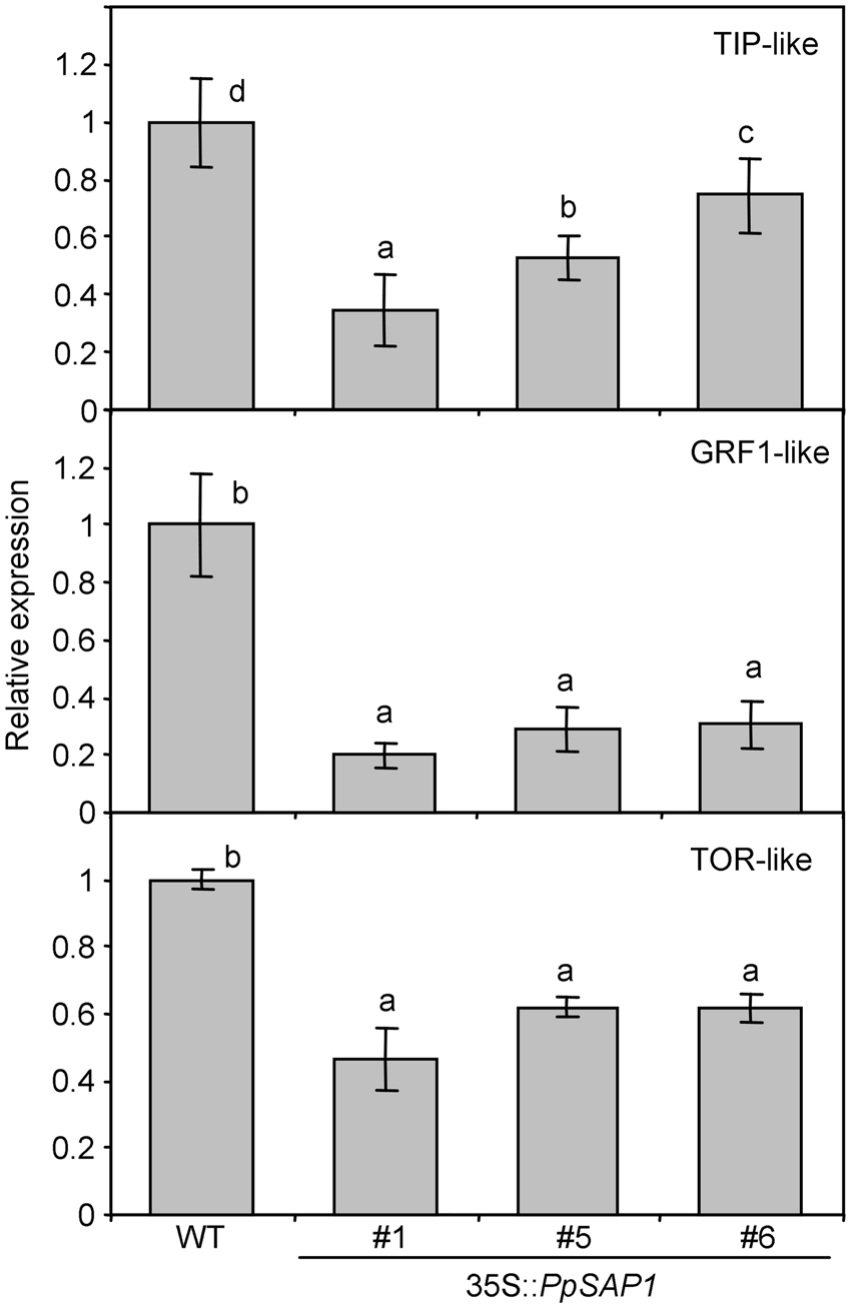
Genes differentially expressed in *PpSAP1* overexpressing lines. The relative expression of *TIP*-like, *GRF1*-like and *TOR*-like genes in wild type (WT) and 35S::*PpSAP1* lines #1, #5 and #6 is shown. An expression value of one is assigned to the WT. Data are means from three biological samples with two technical replicates each, with error bars representing standard deviation. Different letters (**a**–**d**) indicate significant difference between samples with a confidence level of 95%.

Three of those genes were found to be down-regulated by qRT-PCR in plants overexpressing *PpSAP1* (Fig. 9), while other candidate genes did not show a significant variation (Supplementary Fig. S4). *TONOPLAST INTRINSIC PROTEIN* (*TIP*)-like, and *GROWTH-REGULATING FACTOR* (*GRF*)1-like are putative orthologs of genes regulated by the overexpression of *OsiSAP1* in rice^16^, whereas *TARGET OF RAPAMYCIN* (*TOR*)-like is related to a cell growth gene described in *Arabidopsis* and other species^24^.

**Figure 10:**
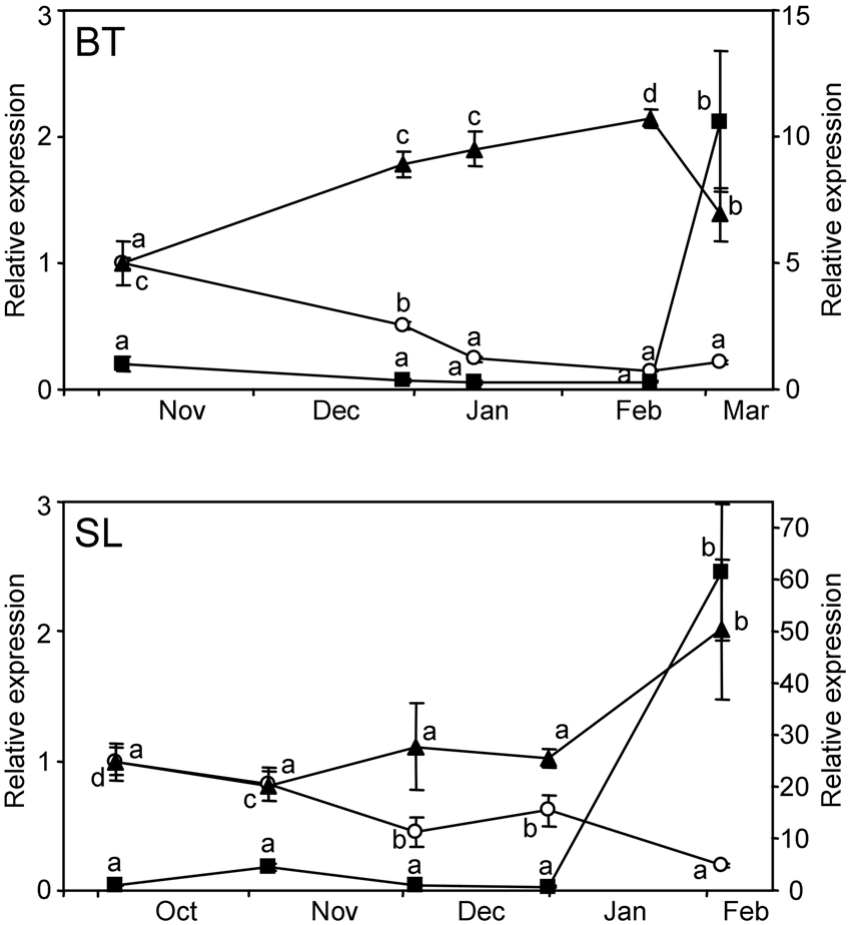
Expression of *TOR*-like and *TIP*-like genes in reproductive buds of peach. The relative expression of *PpSAP1* (white circles, left y-axis), *TOR*-like (black triangles, left y-axis) and *TIP*-like genes (black squares, right y-axis) was measured along bud development in cultivars ‘Big Top’ (BT) and ‘Springlady’ (SL). Dormancy was released in March (BT) and February samples (SL). An expression value of one is assigned to the first sample. Data are means from two biological samples with three technical replicates each, with error bars representing standard deviation. Different letters (**a**–**d**) indicate significant difference between samples for each gene, at a confidence level of 95%.

These results prompted us to examine the expression of *TIP*-like and *TOR*-like genes in the species (peach) and tissue (flower bud) in which *PpSAP1* was first identified. Interestingly, the decrease in *PpSAP1* expression along seasonal bud development correlated well with a quantitatively similar increase in *TOR*-like expression in two independent experiments using two distinct cultivars (Fig. 10). However, the expression of *TIP*-like gene was essentially constant along bud development until it burst in samples collected on February in ‘Springlady’ and March in ‘Big Top’ (Fig. 10). Based on previous physiological measurements^4, 5^, such burst of *TIP*-like expression occurred in dormancy-released buds, while dormant buds only showed a basal level of expression.

## Discussion

Classification of PpSAP1 as a stress-associated protein has taken into account the presence of A20 and AN1 Zn-finger domains and its phylogenetic closeness to described SAP proteins from rice and *Arabidopsis* ^25, 26^, but also molecular and functional issues. *PsSAP1* gene expression was slightly but significantly affected by abiotic stresses such as cold, heat, drought and salinity, in flower buds of peach. In addition, heterologous expression of *PpSAP1* improved retention of water under drought stress in transgenic plum, which resembles increased tolerance to different abiotic stresses conferred by overexpression of *SAP* genes in other species^9, 12–15^.

Relatively little is known about the molecular mechanism of SAP proteins, but some animal and plant counterparts have been postulated to regulate protein stability and regulation by related ubiquitination pathways. In animals, A20 protein performs deubiquitinase and E3 ubiquitin ligase activities to regulate nuclear factor κB signalling^27^, and ZNF216 plays a role on muscle atrophy through the ubiquitin-proteasome system^28^. In plants, both *Arabidopsis* AtSAP5 and rice OsiSAP7 regulate abscisic acid (ABA) signalling and show E3 ubiquitin ligase activity *in vitro* ^10, 11, 18^. In summary, sequence analysis, expression profile and functional characterization of PpSAP1 contributed to categorize it into the SAP group of ubiquitin-binding regulators.

*PpSAP1* is preferentially expressed in peach organs and tissues undergoing dormancy such as bud and embryo, but also in adult leaves. Its expression decreases along flower bud development and embryo cold stratification^29^, which could point to a role of *PpSAP1* in dormancy setting-up and maintenance. *PpSAP1* expression is not strictly linked to the dormancy status of buds, as illustrates the early drop of *PpSAP1* expression depicted in Fig. 1b; however a role of *PpSAP1* in dormancy regulation and meristem growth resumption can not be rule out, as discussed below.

Under overexpression, *PpSAP1* exerts a low but significant effect on water retention in stressed leaves and plants, which supports a potential role of *PpSAP1* in drought tolerance in vegetative tissues and plant organs experiencing developmental dormancy, such as buds and embryos. This becomes particularly meaningful in buds of temperate perennial plants, which have to cope with seasonal environmental constraints such as low temperature and drought stress. Unexpectedly, *PpSAP1* overexpressing plants had an additional morphological phenotype affecting the size and form of leaves. Leaves were smaller and narrower, with an acute leaf base angle, leading to an elliptical shape instead of the most habitual ovate one. Thus, as a consequence of *PpSAP1* overexpression plum leaves became somehow more similar to peach leaves. This smaller leaf size was concomitant with and most likely a result of decreased cell size. The rice *SAP* genes *OsDOG* and *ZPF185* were previously described as suppressors of cell growth by a gibberellin-mediated mechanism^30, 31^. However, contrarily to *PpSAP1*, *ZPF185* expression increased sensitivity to abiotic stresses, suggesting that *SAP* roles on stress and developmental processes are unexpectedly diverse.

Observed water retention and anatomical phenotypes were similar in overexpressing lines #1, #5 and #6, in spite of their different *PpSAP1* expression level (Fig. 5). This could be explained by a saturating effect of *PpSAP1* accumulation on those measurements; or alternatively the effective amount of PpSAP1 protein could be similar in the three transgenic lines, regardless of their distinct *PpSAP1* transcript expression values.

The expression of putative orthologs of genes affected by the overexpression of *SAP*-like genes in rice and *Arabidopsis* ^10, 16, 19^, in addition to several stress-related genes differentially regulated in peach buds were investigated in *PpSAP1* transgenic lines. *GRF1*-like genes were down-regulated in both transgenic rice and plum as a consequence of *OsiSAP1* ^16^ and *PpSAP1* expression, respectively. GRF transcription factors are important regulators of plant growth and development affecting the response to abiotic stresses and leaf morphology and size, among other processes^32^. Interestingly, a triple insertional mutant of *AtGRF1*-*AtGRF3* showed smaller leaves due to a decrease in cell size^33^, resembling the phenotype observed in *PsSAP1* overexpressing lines. These data present *GRF1*-like as a putative transcriptional target of PpSAP1 regulatory pathway with presumable impact on the stress response and cell growth effects described in *PpSAP1* transgenic plants.

On the other hand, *TIP*-like orthologs were differentially regulated in *OsiSAP1*-expressing rice (up-regulated) and *PpSAP1*-expressing plum (down-regulated), which points towards diverging roles and mechanisms of related members of the *SAP* family. TIP aquaporins are involved in water permeability and transport of small molecules across the tonoplast membrane, impinging on stress responses and cell turgor-driven growth^34, 35^. In fact, γ-TIP expression in *Arabidopsis* correlates with cell enlargement^36^, and is increased by gibberellins^37^.

*PpSAP1* overexpression in plum also reduced the expression of *TOR*-like gene, a key regulator of cell growth and metabolism in eukaryotic species in response to nutrient and stress related cues^38^. *TOR* was essential for embryo development in *Arabidopsis* ^39^, and inhibition of TOR function with rapamycin impaired plant growth and development. Most relevantly to this study, suppression of TOR signalling reduced cell elongation in the hypocotyl and led to smaller leaves due to decreased cell size^40^.

**Figure 11:**
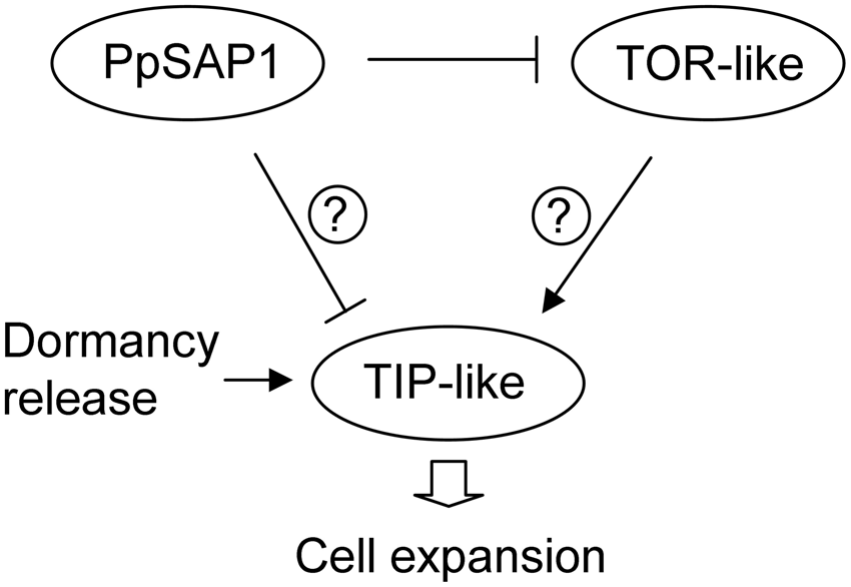
Proposed model of transcriptional interactions between *PpSAP1*, *TOR*-like and *TIP*-like. Transcriptional activation is labelled with an arrow. Transcriptional repression is labelled with a T-shaped line.

*PpSAP1* and *TOR*-like showed opposite expression profiles not only in *PpSAP1* overexpressing lines, but also in flower buds of peach along development (Fig. 10). These data strongly support *TOR*-like repression by PpSAP1 activity or by PpSAP1 downstream effectors (Fig. 11). However, *TIP*-like expression did not correlate with *PpSAP1* accumulation in buds; *TIP*-like expression only peaked in late bud samples, precisely after dormancy release, which suggests that an additional unknown factor may link dormancy release with *TIP*-like expression. The transport of water and other molecules into the tonoplast performed by TIP-like aquaporins would contribute then to increase the cell turgor required for cell expansion and growth, leading to bud-break after the integration of different environmental and intrinsic signals (Fig. 11).

## Methods

### Plant material

The peach plants required for this study were grown at the Instituto Valenciano de Investigaciones Agrarias (IVIA) located in Moncada (Spain). For tissue-dependent gene expression analysis, reproductive and vegetative buds (collected on 12 November 2009), leaves (6 November 2012), embryos, flower parts (26 March 2010) and fruit tissues (29 Juny 2010) were obtained from cv. ‘Big Top’. Reproductive buds of peach were obtained from ‘Springlady’ and ‘Big Top’ cultivars. Collection dates and dormancy status of these buds have been described in detail previously^4, 5^. The effect of stresses on gene expression in peach was studied in dormant buds (collected on 3 November 2015) and dormancy-released buds (25 January 2016) of cv. ‘Crimson Baby’, and non-dormant buds of cv. ‘Rose Diamond’ collected on 2 February 2013. For gene expression analysis of peach leaves under drought stress, leaves gathered from three different trees of cv. ‘Red Candem’ were collected on 27 April 2015. Finally, for leaf discs assays, leaves from five different trees of cv. ‘Big Top’ were collected on 9 June 2015. The culture chamber was maintained at 24 °C with 12 h:12 h light (3 klx):dark photoperiod.

### Stress treatments

To analyze *PpSAP1* expression in flower buds under stress conditions, six budsticks from three different trees per time and treatment were collected. Budsticks were placed in glass tubes with 25 ml of water. Temperature incubations were made at 37 °C, 25 °C (control) and 4 °C in the dark. For salt-stress treatment budsticks were watered with 200 mM NaCl solution, and desiccation stress was carried out in the absence of water. Routinely the base of the budsticks was cut and the solution replaced with fresh one. Flower buds were gathered at 24 h and 72 h.

Stress experiments were also performed on leaf material. For desiccation stress, leaves from three different trees were collected and placed into glasses with the petiole in contact with water (control) or without water (stressed samples), for one, three and seven days. For temperature, salt and ABA treatments, leaf discs were used as described previously^41^. Discs (1 cm diameter) were immersed in a solution containing 5 mM HEPES, 1.5 mM CaCl_2_, and 10 mM KCl during 4 h with gently shaking. After incubation, discs were transferred to fresh solution at 37 °C, 25 °C (control) and 4 °C in the dark for temperature stress. For salt and ABA incubations, discs were transferred and submerged in fresh solutions with 250 mM NaCl and 50 μM ABA, respectively. Ten discs per treatment were collected at 4 h and 24 h. As a control, the expression of a *LEA*-like gene was monitored in leaf samples.

### Isolation of RNA and qRT-PCR

Total RNA from peach material was isolated using the RNeasy Plant Mini Kit (Qiagen, Valencia, CA, USA). Polyvinylpyrrolidone (PVP-40) 1% (w/v) was added to the kit extraction buffer before use. RNA from transgenic plum material was isolated using a rapid cetyltrimethylammonium bromide (CTAB)-based procedure^42^. In both cases, contaminant genomic DNA was removed with the RNase-Free DNase Set (Qiagen) according to manufacturer’s instructions. Total RNA (500 ng) was reverse transcribed with PrimeScript RT reagent kit (Takara Bio, Otsu, Japan) in a final volume of 10 μl. Two μl of a 20X-diluted first-strand cDNA was used for PCR in a total volume of 20 μl. Quantitative RT-PCR was performed on a StepOnePlus Real-Time PCR System (Life Technologies, Carlsbad, CA, USA), utilizing SYBR premix Ex Taq (Tli RNaseH plus) (Takara Bio). The PCR protocol consisted of 10 min at 95 °C, followed by 40 cycles of 15 s at 95 °C, and 1 min at 60 °C. Specificity of the amplification was evaluated by the presence of a single peak in the dissociation curve after PCR and by size estimation of the amplified product by electrophoresis.

Actin-like, AGL26-like, SAND-like and tubulin-like transcripts were used as optional reference genes. Determination of the most stable housekeeping genes was performed using Bestkeeper^43^, NormFinder^44^ and ΔCt^45^. For each group of samples (tissues, bud development and stresses), the genes with better stability value following these three methods were selected as reference genes (Supplementary Table S3). SAND-like was selected as the most stable gene for stress assays and, along with actin-like, for expression analysis of reproductive buds of peach. For tissue expression analysis, actin-like and tubulin-like were the most suitable reference genes. Finally, we used actin-like and AGL26-like for expression experiments in the transgenic plum lines. When two reference genes were required for the analysis, the normalization factor was calculated by the geometric mean of the values of both genes. Relative expression was measured using a relative standard curve. Results were the average of two or three independent biological replicates, with 2–3 technical replicates each. The primers used in this study are listed in Supplementary Table S1.

### Cloning of *PpSAP1* and plasmid construction

For cloning of *PsSAP1* into the yeast two-hybrid plasmid pGBKT7, the whole coding region of *PpSAP1* was PCR-amplified from cDNA obtained from dormant buds of peach, using the primers listed in Supplementary Table S1 under the following PCR conditions: 5 min at 95 °C, followed by 5 cycles of 30 s at 95 °C, 30 s at 57 °C and 1 min at 68 °C, then 30 cycles of 30 s at 95 °C, 30 s at 69 °C and 1 min at 68 °C, and finally 10 min at 68 °C. The PCR product was digested with *Eco*RI and *Bam*HI enzymes and cloned between the *Eco*RI/*Bam*HI restriction sites of pGBKT7 vector (Clontech-Takara Bio).

In order to clone *PsSAP1* into the pROK2 plasmid for constitutive expression of the gene in transgenic plum, *PsSAP1* was amplified using pGBKT7-*PpSAP1* as DNA template with primers listed in Supplementary Table S1. The PCR protocol consisted of 5 min at 94 °C, followed by 3 cycles of 30 s at 94 °C, 30 s at 60 °C and 1 min at 72 °C, then 22 cycles of 30 s at 94 °C, 30 s at 65 °C and 1 min at 72 °C, and a final step of 5 min at 72 °C. The PCR product was digested with *Xba*I and *Bam*HI enzymes and cloned between the *Xba*I/*Bam*HI sites of pROK2 plasmid^46^.

### Phylogenetic analysis of PpSAP1 protein

Sequences similar to *Arabidopsis thaliana* SAP proteins were obtained from *Prunus persica* genome database (https://phytozome.jgi.doe.gov/) through BLASTN search (default parameters, BLOSUM62 comparison matrix), and checked for the presence of both A20 and AN1 domains using the Simple Modular Architecture Research Tool^47^ (SMART; http://smart.embl-heidelberg.de/). Predicted protein sequences were aligned together with PpSAP1 and SAP proteins described in *Arabidopsis* and rice^25^ using Clustal Omega^48^ (http://www.ebi.ac.uk/Tools/msa/clustalo/). A phylogenetic tree was elaborated using Maximum Likelihood method (Bootstrapped with 1000 replicates). Both alignment and phylogeny were carried out in MEGA version 6^49^.

### Analysis of protein interaction by yeast two-hybrid system

The pGBKT7-*PpSAP1* plasmid expressing a fusion of PpSAP1 protein with the Gal4 DNA-binding domain (BD) was introduced into the *Saccharomyces cerevisiae* strain Y2HGold following the yeast transformation procedure and solutions included within the Matchmaker Gold Yeast Two-Hybrid System (Clontech-Takara Bio). The pGBKT7-*PpSAP1* plasmid did not activate autonomously the protein interaction reporters in minimal medium supplemented with the antibiotic Aureobasidin A (AbA) and the chromogenic substrate X-α-Gal at the recommended concentrations (Clontech-Takara Bio).

A yeast two-hybrid library was performed in pGADT7-Rec vector expressing a fusion with the Gal4 activation domain (AD), following the Make Your Own “Mate & Plate^TM^” Library System (Clontech-Takara Bio). Briefly, one μg of total RNA obtained by pooling RNA from dormant and dormancy-released flower buds of peach (‘Big Top’) was reverse transcribed, and cDNA was cloned into pGADT7-Rec by *in vivo* recombination in the yeast strain Y187, following the manufacturer’s instructions. The library contained about 1.5 × 10^6^ independent clones. After mating of Y2HGold strain harbouring pGBKT7-*PpSAP1* with the Y187 library, approximately 3.5 × 10^7^ clones were screened. Two-hybrid interactions were tested in minimal medium without histidine and adenine, and supplemented with AbA (125 ng/ml) and X-α-Gal (40 μg/ml). The inserts contained into positive colonies were amplified using the Matchmaker Insert Check PCR Mix 2 (Clontech-Takara Bio), and digested with *Alu*I and *Rsa*I restriction enzymes for the identification of repeated clones with identical restriction patterns. Independent clones were rescued from yeast using the Easy Yeast Plasmid Isolation Kit (Clontech-Takara Bio), transformed into *Escherichia coli* and sequenced. The protein interaction was confirmed by subsequent transformation of Y2HGold containing pGBKT7-*PpSAP1* with positive clones.

### Genetic transformation of plum

*Agrobacterium tumefaciens* preparation and transgenic plant regeneration of plum (*Prunus domestica* cv. Claudia Verde) were performed according to a previous report^23^. The *Agrobacterium tumefaciens* strain LBA4404, carrying the binary vector pROK2-*PpSAP1* was used. The construction contained the neomycin phosphotransferase gene (*npt*II) for aminoglycoside antibiotic selection of the transgenic plants. For co-cultivation, a 10 ml overnight culture of *Agrobacterium tumefaciens* with an optical density (OD) at 600 nm of 0.2–1.0 was centrifuged at 5,000 × g for 10 min and resuspended in 50 ml of bacterial resuspension medium consisting of MS salts, 2% (w/v) sucrose and 100 µM acetosyringone. This culture was shaken (175 rpm) at 25 °C for 5 h before use.

For plant explant preparation, the endocarp was removed with a nutcracker, and the seeds were surface-sterilized for 30 min using 1% sodium hypochlorite solution containing 0.02% of Tween-20 and rinsed three times with sterile distilled water. Disinfected seeds were soaked in sterile distilled water overnight at room temperature and the seed coats were removed with a scalpel. The radicle and the epicotyl were discarded, and the hypocotyl was sliced into several cross sections (less than 1 mm), which were used for co-transformation.

After 3 days of slice co-culture on shoot regenerating medium (SRM: ¾ MS based medium with 7.5 µM thidiazuron (TDZ), 0.25 µM indole butyric acid (IBA), 9.05 µM 2,4-D and 100 µM acetosyringone), the hypocotyl slices were transferred to SRM selective medium without 2,4-D and acetosyringone, and containing timentin (600 mg/l) and kanamycin (80 mg/l) for 8 weeks. Regenerated shoots were transferred to the shoot growing medium (SGM), in which TDZ was replaced with 1.0 µM 6-benzylaminopurine (BAP).

Plum shoots were maintained by sub-culturing at 4-week intervals on the selective SGM at 23 °C under cool white fluorescent tubes (1.5 klx) and a 16-h photoperiod. When shoots reached 2–3 cm long they were separated from the cluster and transferred to rooting media (RM)^50^ supplemented with kanamycin (40 mg/l). In 3–4 weeks roots started appearing and after 1–3 more weeks, shoots were ready for acclimatization. *In vitro* plants were removed from culture pots and transplanted into pots containing sterilized topsoil sand (4:1) mixture. Plants were covered with transparent plastic pots and progressively removed as plants hardened-off. Control plants were subjected to the same *in vitro* techniques that transformed plants.

### Southern analysis

About 20 µg of *Hind*III- and *Eco*RI-digested genomic DNA samples were separated on 1% (w/v) agarose gels and transferred to positively charged nylon membranes (Roche Diagnostics Corporation, Indianapolis, USA) by capillary blotting. A 696-bp PCR fragment of *npt*II gene was labelled with digoxigenin (DIG) using the PCR DIG labeling mix (Roche Diagnostics Corporation) and the specific primers previously used^51^. Prehybridization and hybridization of blots to the labelled probe were performed at 42 °C. The blots were then washed twice at 23 °C in 2x SSC (0.3 M NaCl, 0.03 M sodium citrate) plus 0.1% (w/v) sodium dodecyl sulfate (SDS) for 15 min, and twice at 65 °C in 0.5x SSC, 0.1% SDS for 15 min. Hybridizing bands were visualized with anti-DIG antibody-alkaline phosphatase and CDPStar (Roche Diagnostics Corporation) on X-ray films.

### Evaluation of water loss and drought tolerance

The water loss under drought conditions was evaluated in detached leaves and whole plants. In the first experiment, we used leaves from plum plants six months after acclimatization. Two leaves from the medium part of the plant were detached from the WT and the transgenic lines #1, #5 and #6, with seven independent plants each genotype. Leaves were dried out on trays at the growth culture chamber conditions. Leaf weight was measured at 30–60 min intervals, for a total time of 340 min.

To determine drought tolerance in transgenic plum plants, two months acclimatized plants were exposed to dehydration stress by stopping watering. After seven days of stress, the fresh weight, weight after rehydration and dry weight of all the plant leaves were measured. The experiment was made with the three different transgenic lines, with twelve plants each line.

The relative water content (RWC) was measured by using the following formula:

$${\rm{RWC}}( \% )=100\ast ({\rm{FW}}-{\rm{DW}})/({\rm{TW}}-{\rm{DW}})$$

where FW is fresh weight, DW is dry weight and TW is turgid weight (the initial leaf weight in the experiment of detached leaves, and the leaf weight after rehydration in the whole plant experiment).

### Morphological and cell size measurements

Morphological measurements were made to wild type and the three transgenic lines (twelve plants each line) two months after acclimatization. To determine leaf area and base angle, photographed leaves were analyzed using ImageJ (version 1.49v, Wayne Rasband, National Institutes of Health, USA).

For cell size measurements, two medium leaves of each plant were collected. After harvest, thin sections of epidermis leaf were excised from the middle part of the leaf, immersed in water, observed using a Leica CTR Mic microscope, and analyzed using ImageJ. Final measurements are the average of 300 cells from ten plants of each line.

### Similarity searches

In order to identify putative orthologs of rice and *Arabidopsis* genes in peach we performed a reciprocal BLASTP analysis. First we made a BLASTP similarity search (https://phytozome.jgi.doe.gov/) on peach database using selected proteins from *Arabidopsis* and rice as queries. The best hit in peach was subsequently compared by BLASTP with *Arabidopsis* or rice databases, and those genes obtained reciprocally by both searches were considered as putative orthologs.

### Statistical analysis

Statistical analyses were performed using the Statgraphics XVI.I package (Statpoint Technologies, Warrenton, VA, USA). Previously, data were evaluated for homoscedasticity and normality in order to select parametric or non-parametric tests. The means of two samples were compared using a Student t-test and comparisons of multiple samples were evaluated by the parametric Fishers’s least significant difference (LSD) test and non-parametric Klustal-Wallis test, with a confidence level of 95% or 99%. Significantly different samples were labelled with asterisks or different letters.

## Electronic supplementary material

Supplementary information
