# Supplementary material for: Dual regulation of water retention and cell growth by a stress-associated protein (SAP) gene in Prunus
Source: Sci Rep. 2017 Mar 23;7:332. doi: 10.1038/s41598-017-00471-7 (PMC5428470; doi:10.1038/s41598-017-00471-7)
Supplement: Supplementary file 1 — Supplementary information [file 41598_2017_471_MOESM1_ESM.pdf]

Dual regulation of water retention and cell growth by a *stress-associated protein* (*SAP*) gene in *Prunus*
A. Lloret, A. Conejero, C. Leida, C. Petri, F. Gil-Muñoz, L. Burgos, M.L. Badenes, and G. Ríos

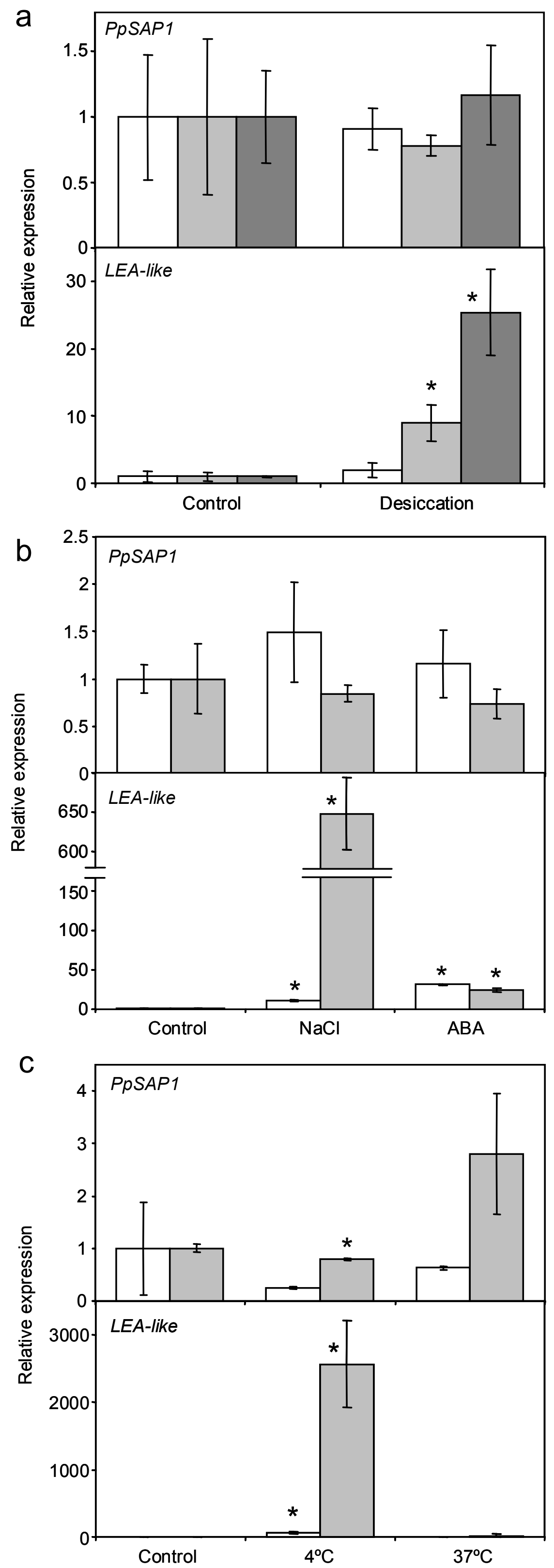

Figure S1. *PsSAP1* gene expression in detached leaves and leaf discs under abiotic stresses. In a, detached leaves were desiccated for one (white bars), three (light grey bars) and seven days (dark grey bars). In b and c, leaf discs were treated for four (white bars) and 24 hours (grey bars). An expression value of one is assigned to the control. *LEA*-like gene (ppa008651m) expression is included as a positive control of stress response. An asterisk indicates significant difference with the control with a confidence level of 95 %.

ppa005503m
GAGCTCGGACACCATTGACAATGTGAAGGCGAAGATCCAGGACAAGGAGGGAATCCCACCAGACCAGCAGAGGTTGATC
TTTGCAGGGAAGCAGCTGGAGGACGGCCGTACTTTGGCTGACTACAATATTCAGAAGGAGTCTACCCTTCACCTTGTGC
TGCGTCTGAGGGGTGGTATGCAGATCTTTGTGAAGACCTTGACTGGAAAGACCATCACGTTGGAGGTGGAGAGCTCGGA
CACAATCGACAATGTTAAGGCCAAGATCCAGGATAAGGAAGGTATTCCCCCAGACCAGCAGAGGTTGATCTTTGCTGGT
AAGCAGTTGGAGGATGGAAGGACTCTGGCCGACTATAACATCCAGAAGGAGTCTACCCTTCACCTTGTCCTCCGTCTCC
GTGGTGGTTTCTAGGAAGTGGAAGTCTCTGGTGTCACCCTGCTTTTAATGAATGTTTCTGTGGTTCCCTTAAAACAATG
TTTTTAAGTATGTTTTTTTAAGTTTCAAGTTTCATGGTACTGTTTCTGCCTCTTGGGGAGGCATTTATCTAGTTTGGTT
TCCTGGTACTTTTTGTTTAATAAATAAATTTGTTCTTGAATAAAAAAAAAAAAAAAAAAAAAAAAAAAA

ppa009116m
GAAGACCCTTACAGGGAAGACAATAACTTTGGAGGTTGAGAGCTCTGACACTATTGACAATGTCAAAGCTAAGATTCAA
GACAAGGAGGGGATCCCACCGGACCAGCAGAGGTTGATCTTTGCCGGGAAACAGCTTGAGGATGGTCGTACCCTTGCAG
ATTATAATATCCAGAAAGAGTCTACTCTCCATCTTGTCCTTCGTCTGCGTGGTGGTGATTTCTGAGTTAGTCCGTTGGT
TTTAGTTAGTTTGTTGGTTATAGTTTGTGTTCTCTAGTGATGGTGGTGAGATGATTATGGTGGTGTGTTGTGTTGTGTT
GATTGGCCGGAGGGCCAATTTAGTA

ppa005507m
ACAAGGAGGGTATTCCCCCAGACCAGCAGAGGTTGATCTTTGCAGGCAAGCAGCTCGAGGATGGAAGGACCCTTGCTGA
CTATAACATTCAAAAGGAGTCCACTCTGCACTTGGTGCTCCGGCTGCGTGGTGGTATGCAGATATTTGTGAAAACTCTG
ACCGGGAAAACCATTACTCTGGAAGTGGAAAGCTCTGATACTATTGATAATGTTAAGGCCAAAATCCAGGACAAGGAGG
GTATTCCCCCAGACCAGCAGAGGTTGATCTTTGCAGGCAAGCAGCTCGAGGATGGAAGGACCCTTGCTGACTACAACAT
TCAAAAGGAGTCCACTCTGCACTTGGTGCTTCGACTGCGTGGTGGGATGCAGATCTTTGTGAAGACTCTGACTGGCAAG
ACCATAACGTTGGAGGTGGAGAGTTCGGATACAATTGATAATGTGAAGGCCAAGATTCAGGACAAGGAGGGCATTCCTC
CAGACCAGCAGAGGCTCATCTTTGCCGGTAAGCAGCTCGAGGATGGGAGGACTCTTGCAGATTACAACATTCAGAAGGA
GTCTACCCTTCACCTTGTCCTTCGTCTCCGAGGTGGTCTCTGAATTCTGAATGATCCTTGTGGTTCCATCCTTTTCTGA
TCAgTGAACAGTGGCTTTGTTAAGTATTTTTATTTTTGAATTTTCGTACTTGGCTGATGGATGATGGGTCACACTCAGA
CTATCTAGTTAATAAATAAACATGCTTTGTATGTAAAAAAAAAAAAAAAAA

ppa007117m
GAGTTCTGATACCATTGATAATGTGAAAGCCAAGATTCAGGACAAGGAGGGCATTCCTCCGGACCAGCAGAGGCTCATC
TTTGCTGGGAAGCAGCTTGAGGATGGCCGCACCCTAGCTGATTACAATATCCAAAAGGAGTCAACCCTTCACTTAGTGC
TTCGTCTTCGTGGGGGTATGCAAATCTTTGTCAAGACACTAACTGGGAAGACGATAACCTTGGAGGTTGAAAGCTCGGA
TACCATAGATAATGTCAAGGCAAAAATTCAGGATAAGGAAGGCATTCCCCCTGATCAGCAGAGGCTAATCTTCGCTGGT
AAGCAACTTGAGGATGGCCGCACCCTTGCCGATTATAACATCCAAAAGGAATCAACTCTGCACCTGGTGCTTCGGTTGC
GTGGTGGTATGCAAATCTTTGTGAAAACTTTGACTGGGAAGACGATTACTTTGGAAGTGGAAAGCTCAGATACTATTGA
CAATGTGAAGGCCAAAATCCAGGACAAGGAGGGTATCCCTCCAGACCAGCAGAGGTTGATATTTGCAGGCAAGCAGCTG
GAGGATGGAAGGACCCTTGCCGACTACAATATTCAAAAGGAGTCGACCCTGCACTTGGTGCTCCGTCTGCGTGGTGGGA
TGCAGATCTTTGTGAAGACTCTGACTGGGAAGACGATAACTTTGGAGGTGGAGAGTTCTGATACCATTGATAATGTGAA
AGCCAAGATTCAGGACAAGGAGGGCATTCCTCCGGACCAGCAGAGGCTCATCTTTGCCGGTAAGCAGCTGGAGGACGGG
AGGACTCTTGCAGATTACAACATTCAGAAGGAGTCTACCCTTCACCTTGTCCTTCGTCTCCGTGGCGGTTTCTGAAGTC
TGAATGATGCTTGTGGTTCCATCCTTGTCGGAGCAGTGAGAAGTGACTTTTAAAAAAA

Figure S2. Sequence of yeast two-hybrid clones.

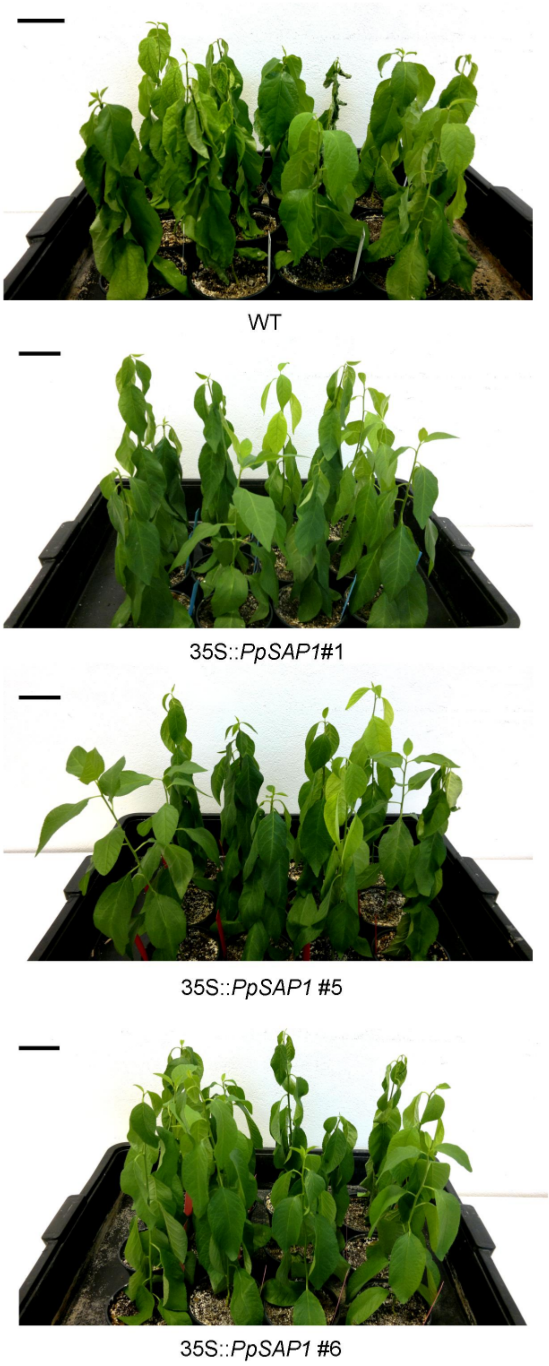

Figure S3. Drought stressed plants for seven days. Two month old plants of WT and transgenic lines 35S::*PpSAP1 #1*, *#5* and *#6* are shown; scale bar, 5 cm.

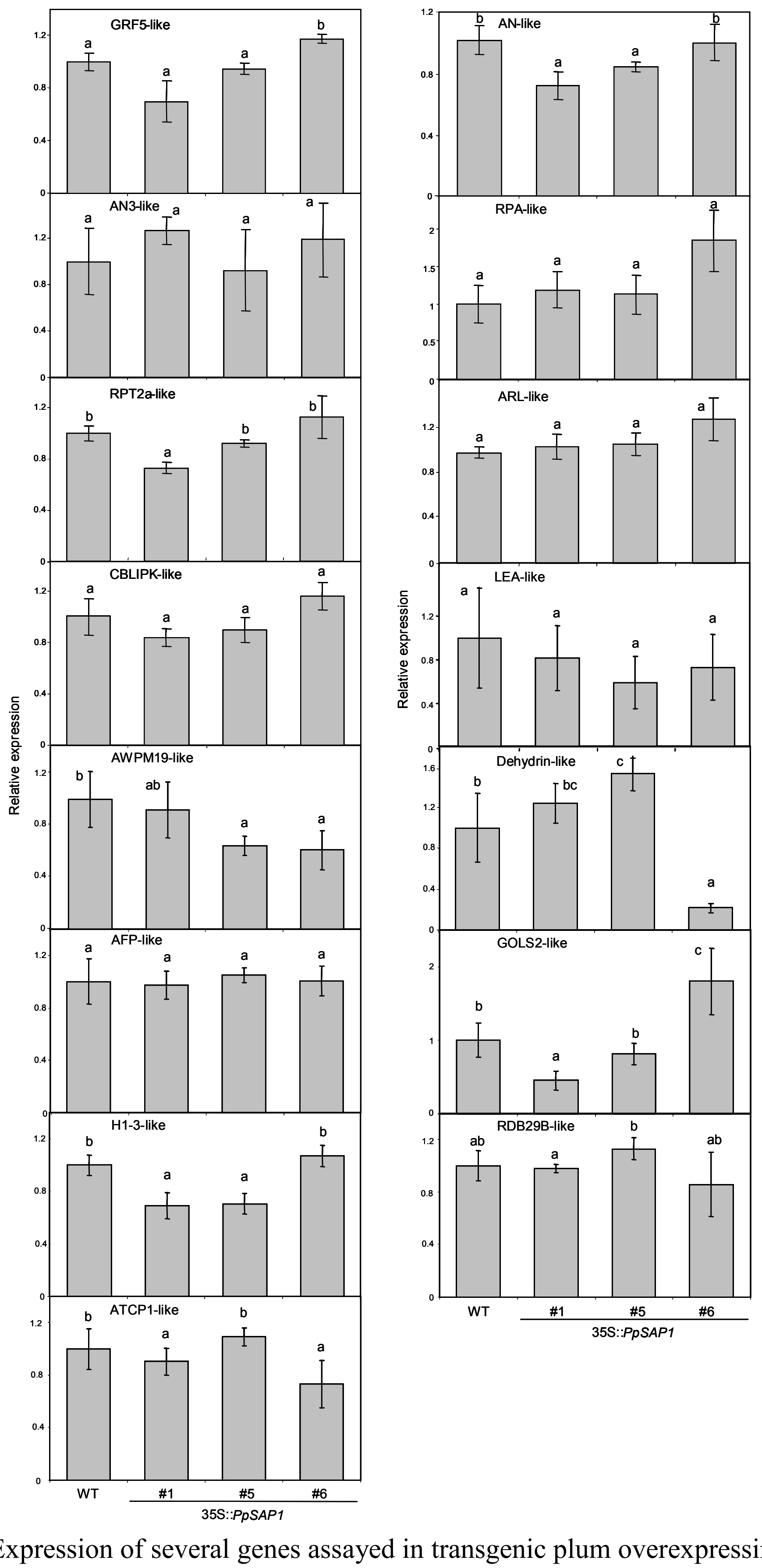

Figure S4. Expression of several genes assayed in transgenic plum overexpressing *PpSAP1*. An expression value of one is assigned to the WT. Data are means from three biological samples with two technical replicates each, with error bars representing standard deviation. Letters (a-b) indicate significant difference between samples.

Table S1. Primers used in this study.

	**Forward**	**Reverse**
**qRT-PCR in peach**		
PpSAP1	ACACAGGCTTCCTCTACTCCATCTTT	GAACCCTCATTCCGAGACATTTATCAG
LEA-like	TCATCTTCCGCTGCCTTTGTAGCCT	GACACTGCCAAGAACACCAAGGACA
Tubulin-like	CAGATGCCCAGTGATGCCTCAG	TGCTTGCCTGATCCAGTCTCAC
AGL26-like	ACCACCTGAAGTCCTCCAAGATTG	GCTTCATACAAAGCAATGCCAACAC
SAND-like	TCGTGGGTACCAGGAAAACGACAT	CCTGCTAGCTTGTGTTCATCTCCA
Actin-like	CTTCTTACTGAGGCACCCCTGAAT	AGCATAGAGGGAGAGAACTGCTTG
**qRT-PCR in plum**		
PpSAP1	TGATCTCAGAGGAGGACCTGCATAT	CAAGTCCTTGTGGCATTTGGAACAC
Plum *SAP1*-like	ACACAGGCTTCCTCTACTCCATCTTT	GAACCCTCATTCCGAGACATTTATCAG
PpSAP1 + plum *SAP1*-like	AACGTGTTGGTCTAACCGGCTTCAAT	TTGTCCAGCTTTTCTGCCTTCACAAC
CBLIPK-like	ACCCCAATGTGGTTCAGCTCTTTGA	AGTAATCAACAGCCCCAACCAACTG
TIP-like	AGTTGTTGCTTGCTTGCTACTCAAG	GCACCAACAATCAAACCAATTGCGA
GRF1-like	GGGGTTCTATCCATTTGGGATTCTC	TTTTTGGTCAGGAACGGCATCTCTC
GRF5-like	GGAGGTGCAGAAGAACAGATGGTAA	TTGAACGGTTTCTGCCTCTGTGCAT
RPA-like	GGGTTGCTAACAAGCAGTTTAAGAC	GGGACCTAACATATCAACTGGAACA
AN-like	AAAGCCTGGTGAGCTGTTGAAGGAT	AGTATCTAGGAACCAACCACCACCT
AN3-like	ATGTACCTGGCTGCCATTGCTGATT	GCTTGTTGCTGCATGTAATGTGCTC
TOR-like	GCAGTACCAAAGAAGATTGGGCAGA	GCAAATAACTCGCGCCCAACAAAT
ARL-like	TCGTCGAACACTATCACACGGTAG	CAACAGAGTCGCCGTGAGACATATA
RPT2a-like	GTGGCAAACTCAACATCTGCAACT	GTGAGAGGTCATCAGCAACCCTAA
AWPM19-like	CCCAGCCAATATGGCGAATATCAGAA	CATAGTGAGCAGCAGTAAGTTTGTGCT
Dehydrin-like	GTACTCTCATGACACCCACAAAACTAC	CCCGGCCCCACCGTAAGCTCCAGTT
AFP-like	TTCCGTTGGTGGTGGAGTGGATGCA	TTACTAGCAGGGCTTCTTGCTTCAC
H1-3-like	AAACCGCTGCTCATCCTCCATACT	TGCTTCTCCTCCATGTACTTGGCT
RD29B-like	ATCTGCTAAGAACGTCGTCGCTTC	GGCTTTGCTGTAACCTCCTGATGA
ATCP1-like	AAGGTTATGGACAAGGACGGGGAT	CATGGCCTTGATGTCTTCATCAGTG
GOLS2-like	TGACCACCTGTTTGACTACCCAGA	GCTGGCAGTACCCAATCTTGTACT
**Two-hybrid cloning**		
	CCAGAATTCATGGAGCACAACGAGACAG	GCCGGATCCTCAGATTTTGTCCAGCTTTTC
**pROK2 cloning**		
	CAGTCTAGATGGAGGAGCAGAAGCTGATCTC	GCCGGATCCTCAGATTTTGTCCAGCTTTTC

Table S2. List of genes tested by qRT-PCR in transgenic plum and peach.

Name	Rice or *Arabidopsis* gene/protein	Peach putative ortholog	Protein similarity	Reference
CBLIPK-like	LOC_Os01g10890	ppa005365m	CBL-interacting protein kinase	(1)
TIP-like	LOC_Os01g74450	ppa010364m	Tonoplast intrinsic protein1-3	(1)
GRF1-like	LOC_Os03g51970	ppa019623m	Growth-regulating factor 1	(1)
GRF5-like	LOC_Os02g53690	ppa017593m	Growth-regulating factor 5	(1)
RPA-like	LOC_Os03g11540	ppa003038m	Replication protein A subunit B	(1)
AN-like	AT1G01510	ppa003091m	Angustifolia	(2)
AN3-like	AT5G28640	ppa011329m	Angustifolia3	(3)
TOR-like	AT1G50030	ppa000022m	Serine/threonine-protein kinase TOR	(4)
ARL-like	AT2G44080	ppa013582m	Argos-like	(5)
RPT2a-like	AT4G29040	Prupe.8G208900	26S proteasome regulatory subunit	(6)
LEA-like		ppa008651m	Late embryogenesis abundant	(7)
AWPM19-like		ppa012188m	AWPM19	(7)
Dehydrin-like		Prupe.7G161100	Dehydrin	(7)
AFP-like		ppa006974m	ABI5 binding protein	(7)
H1-3-like	AT2G18050	ppa011941m	Histone H1-3	(8)
RD29B-like	AT5G52300	ppa001989m	Responsive to desiccation 29B	(8)
ATCP1-like	AT5G49480	ppa012594m	NaCl-inducible calcium-binding protein	(9)
GOLS2-like	AT1G56600	ppa008294m	Galactinol synthase 2	(9)

(1) Dansana, P. K., Kothari, K. S., Vij, S. & Tyagi, A. K. *OsiSAP1* overexpression improves water-deficit stress tolerance in transgenic rice by affecting expression of endogenous stress-related genes. *Plant Cell Rep.* **33**, 1425–1440 (2014).

(2) Kim, G. T. *et al*. The *ANGUSTIFOLIA* gene of *Arabidopsis*, a plant CtBP gene, regulates leaf-cell expansion, the arrangement of cortical microtubules in leaf cells and expression of a gene involved in cell-wall formation. *EMBO J.* **21**, 1267–1279 (2002).

(3) Kim, J. H. & Kende, H. A transcriptional coactivator, AtGIF1, is involved in regulating leaf growth and morphology in *Arabidopsis*. *Proc. Natl. Acad. Sci. U. S. A.* **101**, 13374–13379 (2004).

(4) Menand, B. *et al*. Expression and disruption of the *Arabidopsis TOR* (target of rapamycin) gene. *Proc. Natl. Acad. Sci. U. S. A.* **99**, 6422–6427 (2002).

(5) Hu, Y., Poh, H. M. & Chua, N. H. The Arabidopsis *ARGOS-LIKE* gene regulates cell expansion during organ growth. *Plant J.* **47**, 1–9 (2006).

(6) Kurepa, J. *et al*. Loss of 26S proteasome function leads to increased cell size and decreased cell number in *Arabidopsis* shoot organs. *Plant Physiol.* **150**, 178–189 (2009).

(7) Leida, C. *et al*. Histone modifications and expression of *DAM6* gene in peach are modulated during bud dormancy release in a cultivar-dependent manner. *New Phytol.* **193**, 67–80 (2012).

(8) Giri, J. *et al*. Rice A20/AN1 zinc-finger containing stress-associated proteins (SAP1/11) and a receptor-like cytoplasmic kinase (OsRLCK253) interact via A20 zinc-finger and confer abiotic stress tolerance in transgenic *Arabidopsis* plants. *New Phytol.* **191**, 721–732 (2011).

(9) Kang, M. *et al*. Arabidopsis SAP5 functions as a positive regulator of stress responses and exhibits E3 ubiquitin ligase activity. *Plant Mol. Biol.* **75**, 451–466 (2011).

Table S3. Stability index of reference genes for real-time experiments using three methods.

	NormFinder	BestKeeper	ΔCt
**Tissues**			
Tubulin-like	0.29	1.48	0.91
AGL26-like	0.41	1.91	1.17
SAND-like	0.30	1.61	0.92
Actin-like	0.37	1.39	1.02
**Reproductive buds**			
Tubulin-like	0.19	1.51	0.52
AGL26-like	0.33	1.73	0.64
SAND-like	0.18	1.21	0.46
Actin-like	0.28	1.15	0.58
**Stress assays**			
Tubulin-like	0.45	1.69	1.07
AGL26-like	0.25	1.25	0.75
SAND-like	0.14	1.10	0.60
Actin-like	0.29	1.23	0.76
